# Novel *GJA1/Cx43* Variant Associated With Oculo-Dento-Digital Dysplasia Syndrome: Clinical Phenotype and Cellular Mechanisms

**DOI:** 10.3389/fgene.2020.604806

**Published:** 2021-01-27

**Authors:** Irene Sargiannidou, Violetta Christophidou-Anastasiadou, Andreas Hadjisavvas, George A. Tanteles, Kleopas A. Kleopa

**Affiliations:** ^1^Department of Neuroscience, The Cyprus Institute of Neurology and Genetics, Nicosia, Cyprus; ^2^Clinical Genetics Center, The Cyprus Institute of Neurology and Genetics, Nicosia, Cyprus; ^3^Department of Clinical Genetics, Makarios Hospital, Nicosia, Cyprus; ^4^Department of Molecular Pathology and Electron Microscopy, The Cyprus Institute of Neurology and Genetics, Nicosia, Cyprus; ^5^Center of Neuromuscular Disorders, The Cyprus Institute of Neurology and Genetics, Nicosia, Cyprus; ^6^Center for Multiple Sclerosis and Related Disorders, Cyprus School of Molecular Medicine, The Cyprus Institute of Neurology and Genetics, Nicosia, Cyprus

**Keywords:** connexin43, *GJA1* gene, oculodentodigital dysplasia, gap junctions, leukodystrophy

## Abstract

Oculodentodigital dysplasia syndrome is associated with numerous pathogenic variants in *GJA1*, the gene encoding connexin43 gap junction protein. A novel in-frame deletion (p.Lys134del) was found in our clinic. The patient showed all the typical dysmorphic features of the syndrome. The functional consequences of this variant were also studied in an *in vitro* system. Cells expressed significantly less number of gap junction plaques with a great number of them retained intracellularly.

## Introduction

Oculodentodigital dysplasia (ODDD) syndrome is a rare disease with an estimated prevalence < 1/1,000,000. It is characterized by nystagmus; microphthalmus; microcornea; anteverted nostrils; syndactyly of the third, fourth, and fifth digits; craniofacial deformities; skin abnormalities; and dental anomalies (Paznekas et al., 2003). Radiographs usually reveal hyperplasia of the body of the mandible and broadening of the tubular bones. Several reports have noted spasticity and hyperreflexia as well as white matter changes and calcification of the basal ganglia. Spastic bladder or gait disturbances are the most frequent neurologic presentations starting in the second decade of life (Loddenkemper et al., 2002). ODDD results from autosomal dominant Gap Junction Alpha1 (*GJA1*) gene pathogenic variants encoding connexin43 (Cx43). There are 21 isoforms of connexin family genes in the human genome. Each connexin is composed of four trans-membrane domains, N-terminal domain, C-terminal domain, one intracellular, and two extracellular loops (Sohl and Willecke, 2004). The C-terminal tail of Cx43 is long and highly conserved. It plays a role in regulating pH whereas the N-terminal domain plays a role in the channel’s state between open and closed. The extracellular loops enable the channel docking while the intracellular loop is responsible for Cx43 movement toward the plasma membrane (Nambara et al., 2007). Cx43 is a ubiquitously expressed gap junction (GJ) protein detected in most cell types, including the heart, CNS where it is mostly expressed by astrocytes, bone, testicles, and skin (Laird, 2014). Currently, more than 70 different nonsense and missense variants that give rise to ODDD phenotype have been reported^1^. Reports have also shown that *GJA1* pathogenic variants are associated with heart malfunctions, hearing loss, and skin disorders (Alexandrino et al., 2009). ODDD pathogenic variants are thought to mainly disrupt intercellular communication required for homeostasis and signaling (Delmar et al., 2018). In this study, we describe two related individuals with clinical symptoms of ODDD caused by a novel *GJA1* variant and reveal the underlying cellular mechanisms.

## Methods

### Genetic Testing

Genomic DNA was extracted from blood in EDTA. Analysis was performed by PCR followed by direct sequencing of all the coding regions and splice sites of the *GJA1* gene with GenBank accession no. NM_000165.5 (*GJA1*). Screening of 110 healthy individuals was also performed.

### Cellular Expression of the Novel GJA1 Variant

The mutated *GJA1* gene was PCR-amplified from genomic DNA of patient II following informed consent by the patient under the Protocol “Translational Research-Neurogenetics Department” approved by the Cyprus National Bioethics Committee (EEBK/EP/2013/28), using the following primers: XhoI-Cx43-forward, 5′AAATTTAAACTCGAGAACATGGGTGACTGGA 3′ and BamHI-Cx43- reverse, 5′ AAATTTAAAGGATCCCTA GATCTCCAGGTCAT3′. WT *GJA1* gene was also PCR amplified using the same primers from genomic DNA of a non-diseased patient. Both the PCR amplicons and the pIRES2-EGFP (Clontech) expression plasmid were digested with the XhoI and BamHI enzymes to generate compatible ends and then ligated together. Expression of either the WT Cx43 or the Mutant Cx43 was driven by a CMV promoter. In order to co-express both WT and mutant Cx43 into same cells, the pBudCE4.1 dual vector was used to clone in the Cx43^K134del^ sequence downstream of the CMV promoter and the WT Cx43-IRES-EGFP downstream of the EF-1Fa promoter. Correct assembly of the expression cassettes was confirmed by direct sequencing of the constructs.

### Cell Transfections and Immunofluorescence

HeLa cells were transfected using the cationic lipid-based Lipofectamine LTX with PLUS reagent (Invitrogen). One hundred eighty nanograms of plasmid DNA was used for the DNA–lipid complex and was added dropwise to the cells. Cx43 expression of the cells was examined 48 h post-transfection. Transfected cells were washed with phosphate buffered saline (PBS), fixed in 4% paraformaldehyde (PFA), blocked with 5% bovine serum albumin (BSA), and then were incubated with primary antibodies overnight at 4°C, including rabbit anti-Cx43 (1:50, Cell Signaling), mouse anti-Cx43 (1:200, Millipore), mouse anti-calnexin (1:50, Abcam), and mouse anti-58k (1:50, Abcam). The slides were visualized with a fluorescence microscope using Axiovision software (Carl Zeiss MicroImaging). For quantification of GJ plaque formation, we counted the number of GJ plaques per cell in *n* = 40 cells for each experiment where mutant and WT Cx43 were co-expressed, compared to the number of GJ plaques formed in cells expressing only the WT or the mutant Cx43. GJ plaques were defined as individual concentrations of connexin immunoreactivity measuring between 0.1 and 1 μm^2^. Transfection experiments were performed three times. Statistical significance was assessed by one-way ANOVA followed by Bonferroni’s *post hoc* test. A value of *p* < 0.05 was considered statistically significant.

## Results

### Clinical Findings

Both patients were examined by a clinical geneticist and neurologist. Genetic testing of patient I was done after informed consent was obtained from both parents.

Patient I: This is an 11-year-old girl who was initially evaluated at the age of 3 years old. She was the first female child born to healthy unrelated parents following unremarkable pregnancy without maternal history of concurrent infection or known exposure to teratogens. Antenatal scans were unremarkable. She was born at 38/40 gestation by an emergency LSCS due to failure to progress. Cleft upper lip (non-midline) and 4th–5th finger skin syndactyly was noted at birth. Echocardiography revealed a patent foramen ovale. At the time of initial evaluation, she had typical facial features consistent with ODDD including microphthalmia, a pinched nose with hypoplastic alae nasi, and thin nares. She also had mild speech delay with otherwise normal growth parameters.

Patient II: This 40-year-old woman, mother of patient I, reported tremor and mobility problems since early childhood. She could never keep up with her peers in sports activities. She was also found since early childhood to have dental anomalies requiring multiple procedures for correction. Adhesion of digits 4 and 5 bilaterally in the hands were treated surgically in childhood. She reported learning difficulties throughout her school years. She complained about progressive difficulty walking, impaired balance, and urgency of urination with occasional incontinence. Besides her affected daughter, no other affected family members could be confirmed. Her father was seen by the neurologist and did not have any apparent features of the disease. Her mother died at the age of 38 of unknown etiology and it remains unclear whether she had any features of the syndrome. Examination revealed the typical dysmorphic features of ODDD. Her hearing and vision were intact. She has mild dysarthria, which might be related to the nasal dysplasia. On motor examination she has full muscle power throughout, but fine motor movements were impaired in the hands and feet and she had postural and action tremor with mild dysmetria. There was increased muscle tone with spasticity especially in the lower limbs. Reflexes were brisk throughout. Plantar responses were extensor bilaterally. There was no cerebellar dysfunction. Sensory examination showed reduced pinprick and vibratory sensation in distal limbs. Her Romberg’s test was positive.

**FIGURE 1:**
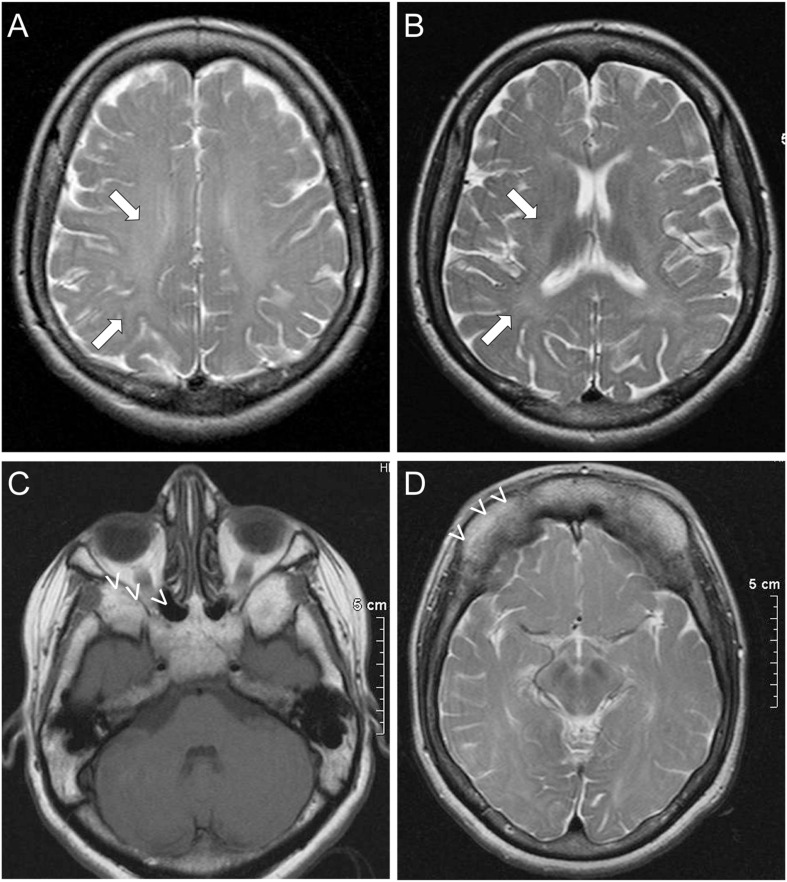
Imaging features of ODDD: Brain MRI of patient II shows subtle findings of leukodystrophy, with delayed myelination in the white matter (arrows) most prominently of the frontal and parietal lobes in the centrum semiovale **(A)** and toward the internal capsules **(B)**. Dysmorphic features of ODDD including hypoplasia of the nasal and maxillary sinuses with small orbits **(C)** and complete aplasia of the frontal sinuses **(D)** are also demonstrated (open arrowheads).

Further investigations in patient II included normal routine hematological and complete biochemistry studies, thyroid function tests, vitamin B12, and folic acid levels. An MRI of the brain, showed evidence of leukodystrophy with delayed myelination of the cerebral white matter (Figures 1A,B). Characteristic dysplastic changes in the facial bones with hypoplasia of the paranasal sinuses, as well as aplasia of the frontal sinuses associated with thickening of the frontal calvarium were also noted (Figures 1C,D). MRI of the spine revealed no spinal cord abnormalities but there were mild degenerative disc and arthritic changes causing a mild degree of stenosis of the lumbar canal. Visual evoked potential studies revealed prolonged absolute P100 latencies bilaterally (left 130 ms, right 134 ms) indicative of optic pathway dysfunction. Brainstem auditory evoked potentials revealed prolonged absolute wave V and I–V and III–V interpeak latencies bilaterally at 80 dB nHL, indicative of bilateral acoustic pathway dysfunction between the pons and midbrain. Finally, both upper as well as lower limb somatosensory evoked potentials revealed prolonged latencies corresponding to somatosensory pathway dysfunction throughout the CNS. A routine EEG revealed no abnormalities. EMG/nerve conduction studies showed no evidence of peripheral neuropathy, but there was evidence of mild chronic right L4–L5 radiculopathies. An echocardiogram revealed no abnormalities and a 24-h 3-Lead Holter monitoring study showed normal sinus rhythm with multiple episodes of sinus tachycardia up to 140/min.

### Genetic Findings

**FIGURE 2:**
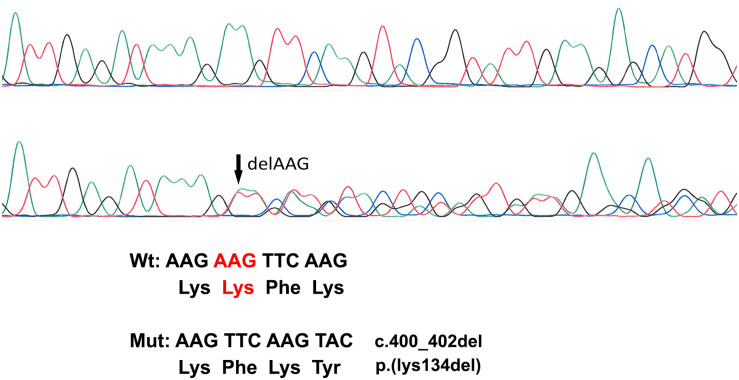
Sanger Sequencing Analysis. The top electropherogram is from the non-affected control DNA sample while the bottom one is from patient I DNA analysis showing a three-amino-acid deletion (c.400_402del) (marked with an arrow).

A novel heterozygous change consisting of a three-nucleotide deletion in exon 2 of the *GJA1* gene (c.400_402_del) was identified both in the proband (patient I) and her mother (patient II), which results in the deletion of the amino acid lysine at codon 134 (p.Lys134del) (Figure 2). This in-frame deletion lies within the intracellular loop of the Cx43 protein. Within the same region, five missense pathogenic variants (p.Ile130Thr, p.Lys134Glu, p.Lys134Asn, p.Gly138Ser, and p.Gly138Arg) have been previously reported (Paznekas et al., 2003). Screening of 110 healthy Cypriot controls as well as search in the gnomAD ver2.1.1 database showed the absence of this variant. According to the ACMG classification system, this variant is likely pathogenic.

### Functional Analysis

**FIGURE 3:**
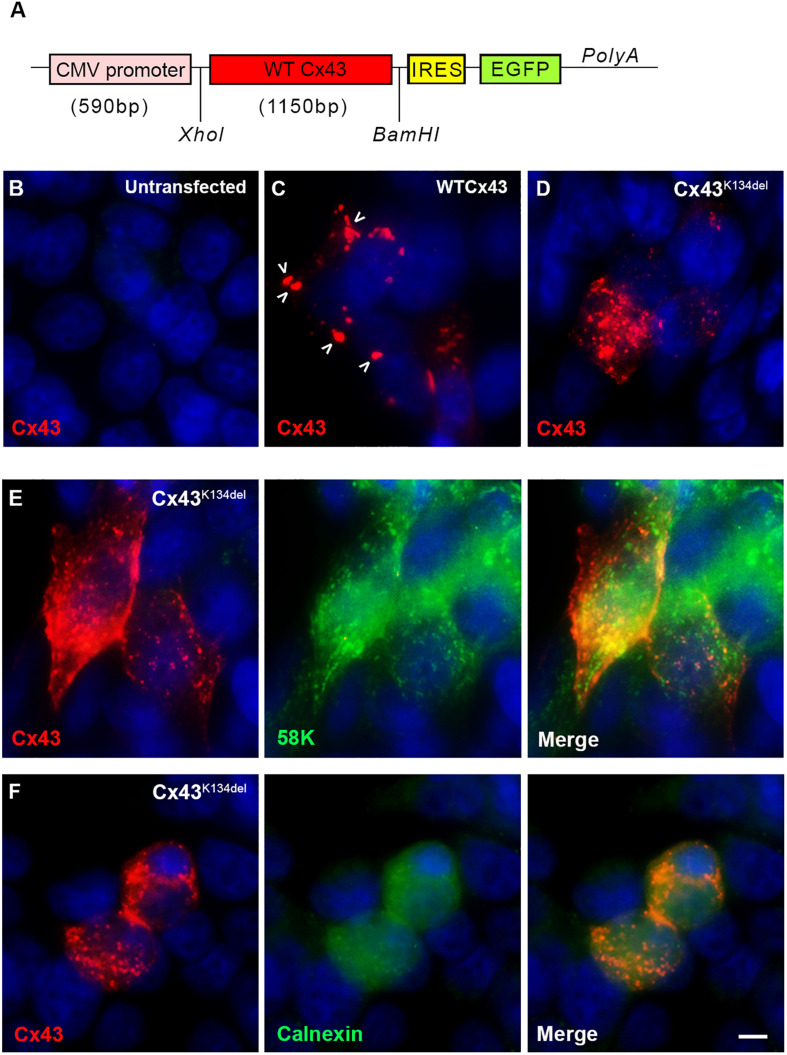
Localization of Cx43^K134del^. Diagram of the expression cassette used in the cell expression studies **(A)**. Immunofluorescence staining for Cx43 (red) in untransfected HeLa cells used as negative control **(B)** or transfected cells expressing either WT Cx43 (normal sized plaques are marked with arrows) **(C)** or the Cx43^K134del^ mutant **(D–F)** reveals that in contrast to the formation of GJ plaques by WT Cx43, the Cx43^K134del^ mutant is localized intracellularly **(D)** in the ER-Golgi compartment as confirmed by further double staining with either the Golgi marker 58k **(E)** or the ER marker calnexin **(F)** (both in green). Cell nuclei were counterstained with DAPI (blue). Scale bar = 10 μm.

To examine the expression of the mutated Cx43, we transfected HeLa cells with a plasmid that carries either the mutated or the WT Cx43 ORF (Figure 3A). In contrast to untransfected cells (Figure 3B), cells expressing WT Cx43 showed good sized GJ plaques localized on the cell surface (Figure 3C) while cells expressing Cx43^K134del^ showed mostly intracellular and granular Cx43 immunoreactivity with almost complete disruption of membrane GJ plaque formation (Figures 3D–F). In order to evaluate the subcellular localization of Cx43^K134del^, transfected cells they were double labeled with the Golgi marker 58K (Figure 3E) or the endoplasmic reticulum (ER) marker Calnexin (Figure 3F). The mutant Cx43 protein co-localized with both markers indicating intracellular retention in both compartments along the Cx43 biosynthesis pathway.

**FIGURE 4:**
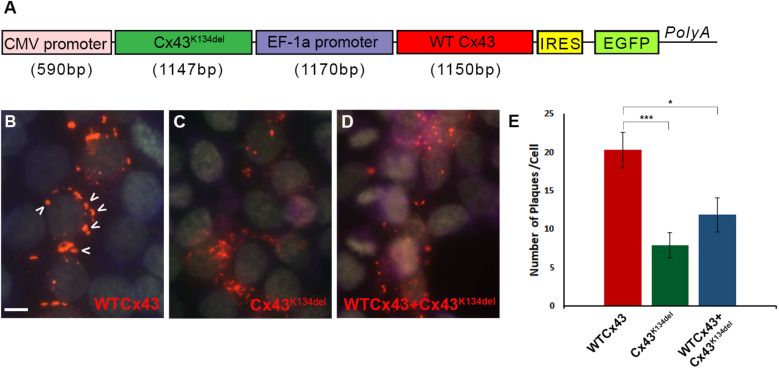
Dominant-negative effects of Cx43^K134del^ on WT Cx43. Diagram of the expression cassette that carries both WT Cx43 and Cx43^K134del^ **(A)**. Representative images of immunostained HeLa cells expressing WT Cx43 (marked with arrows) **(B)** or Cx43^K134del^ **(C)** or co-expressing WT Cx43 along with Cx43^K134del^ **(D)**. Scale bar = 10 μm. Quantification of plaques formed in the transfected cells **(E)** shows significant reduction of the number of plaques formed by Cx43^K134del^ compared to WT Cx43. Importantly, the number of plaques formed on cells expressing WT Cx43 along with Cx43^K134del^ were significantly lower than on the WT Cx43 transfected cells (*p < 0.05, ***p < 0.001, one way ANOVA with Bonferroni *post hoc* test was used). Standard error of the mean (SEM) is shown in the graph.

Finally, in order to examine whether Cx43^K134del^ could interfere with WT Cx43, HeLa cells were transiently transfected with a dual vector that carried both ORFs (Figure 4A) and quantified the expression of Cx43 (Figures 4B–D). The expression of WT Cx43 along with the EGFP reporter gene was driven by the EF-1a promoter while the mutated Cx43 ORF was driven by the CMV promoter. Counts of the number of GJ-like plaques formed on the cell membrane of Cx43^K134del^ transfected cells showed a significant reduction compared to WT Cx43 transfected cells (*p* < 0.001). These plaques were significantly reduced in numbers, but they were also smaller in size. Most of the plaques on the Cx43^K134del^ transfected cells were close to 0.2 μm^2^. Moreover, cells co-expressing WT Cx43 and the Cx43^K134del^ mutant showed significant reduction of GJ plaques formed compared to WT Cx43 alone (*p* < 0.05), indicating a dominant-negative effect of the mutant on co-expressed WT Cx43.

## Discussion

In this report, we describe the first cases of ODDD in Cyprus caused by a novel *GJA1* pathogenic variant and clarify the cellular mechanisms of the disease. Our patients presented with a typical ODDD phenotype as previously reported (Paznekas et al., 2003; De Bock et al., 2013). Intra-familial variability as described in other ODDD families (Loddenkemper et al., 2002; Paznekas et al., 2009) is suggested by the more severe manifestations and earlier diagnosis in the daughter; however, the mother retrospectively presented with similar symptoms in childhood but remained undiagnosed until her daughter came to medical attention. The manifestation of leukodystrophy in patient II is in keeping with other ODDD phenotypes and likely reflects the dysfunction of oligodendrocytes that heavily depend on their GJ connectivity to astrocytes for maintaining homeostasis (May et al., 2013). Cx43 is the main astrocytic partner on oligodendrocyte to astrocyte GJ channels especially in the white matter (Kamasawa et al., 2005; Markoullis et al., 2012, 2014).

This family carries a novel *GJA1* change causing ODDD, which has not been reported before. However, this in-frame deletion lies within the intracellular loop that is known to be the most variable domain across different connexin family members. The intracellular loop along with the carboxy terminus of Cx43 are mediating the pH gating (Duffy et al., 2002). Five missense pathogenic variants, including p.Ile130Thr, p.Lys134Glu, p.Lys134Asn, p.Gly138Ser, and p.Gly138Arg have been reported in the same region in ODDD patients (Paznekas et al., 2003; Richardson et al., 2004).

We further investigated the cellular mechanisms of this novel *GJA1* variant in cultured cells, demonstrating the abnormal cellular expression with retention intracellularly as well as a dominant-negative effect of the Cx43^K134del^ mutant on co-expressed wild-type (WT) Cx43. We show that the novel Cx43 variant caused retention intracellularly in the ER-Golgi compartment and likely exerts dominant-negative effects on co-expressed WT protein decreasing its trafficking to the cell membrane and formation of GJ plaques. However, since the WT and mutant Cx43 were transcribed under the control of different promoters in the same cell, their expression levels could be different. This difference could introduce bias in our experiments and conclusions. Comparison of constitutive promoters has previously shown that the level of expression by these promoters depends on the cell line used and the experimental settings (Qin et al., 2010; Damdindorj et al., 2014). CMV is prone to silencing over time but all our experiments were transient transfections where expression is evaluated 48 h post-transfection, thus eliminating this factor.

Several Cx43 variants have been characterized as to their effect on Cx43 channel functionality. Both G21R and G128R are loss-of-function changes as shown by Roscoe et al. (2005). When frameshift mutation 260 (fs260) was expressed in HeLa cells, ER retention was observed. This pathogenic variant had a dominant-negative effect on including the F52dup, R202H, Y17S, G21R, and A40V that failed to form GJ plaques. The L90V, I130T, and K134E mutants formed GJ channels, but their functionality was compromised when their permeability was evaluated (Shibayama et al., 2005). Patch-clamp analysis of four other mutants, Q94K, L90V, R202H, and V216L, showed reduced conductance (McLachlan et al., 2005). In cases where the pathogenic variants are located in the same compartment of the protein, like the G138R and G143S mutations, the effect on WT Cx43 can be different (Kelly et al., 2016). Even though communication studies would further support the results of our study, they were not performed as our mutant is mostly retained intracellularly and not localized to the cell membrane; thus, it is expected that cell-to-cell communication will be reduced. Our co-expression study provides further evidence not only for the intracellular retention of this Cx43 mutant, but also for its dominant effect on the WT Cx43 likely to be the underlying cellular mechanism of this mostly dominantly inherited disease.

Similar cellular effects have been demonstrated with other connexin mutants associated with other disorders. Cx26 variants associated with hearing loss showed intracellular retention in the ER or in the early endosomes, and their loss of function appears to be due to premature degradation (Xiao et al., 2011; Beach et al., 2020). Several Cx32 mutants causing neuropathy showed complete absence (Sargiannidou et al., 2015) or abnormal intracellular retention in the ER and/or Golgi (Kleopa et al., 2002, 2006), often with dominant effects on co-expressed WT Cx32 with demonstrated direct protein–protein interaction, likely during hexamer formation in the Golgi (Kyriakoudi et al., 2017). Other Cx32 mutants reached the cell membrane but showed shifted voltage gating and abnormally increased opening of the channels (Abrams et al., 2002). Finally, similar to the Cx43 mutants, Cx47 variants associated with leukodystrophy showed retention in the ER and failure to form functional GJ channels (Orthmann-Murphy et al., 2007).

In conclusion, we described the first Cypriot family with ODDD caused by a novel change in *GJA1* gene, presenting the characteristic phenotype of the disease. Moreover, our expression analysis provides further support for the intracellular retention of mutant Cx43 and its dominant-negative effect on the WT allele as an underlying mechanism of the disease. With this work, we proved that the variant is pathogenic, class 5, according to the ACMG classification system.

## Data Availability Statement

The authors acknowledge that the data presented in this study must be deposited and made publicly available in an acceptable repository, prior to publication. Frontiers cannot accept a manuscript that does not adhere to our open data policies.

## Ethics Statement

The studies involving human participants were reviewed and approved by The Cyprus National Bioethics Committee (EEBK/EP/2013/28). Written informed consent to participate in this study was provided by the participants’ legal guardian/next of kin.

## Author Contributions

IS and AH were responsible for experiments and data collection. VC-A, GT, and KK were responsible for patient examination and clinical evaluation data. IS and KK were responsible for manuscript draft and critical revision. All authors approved the final version.

## Conflict of Interest

The authors declare that the research was conducted in the absence of any commercial or financial relationships that could be construed as a potential conflict of interest.

## Footnotes

1 http://www.hgmd.cf.ac.uk/ac/all.php
